# Time Across the Lines: Collaborative Wonderings Under COVID-19

**DOI:** 10.1177/1077800420962476

**Published:** 2021-09

**Authors:** Brook Bolander, Philippa Smith

**Affiliations:** 1 Monash University, Melbourne, Victoria, Australia; 2 Auckland University of Technology, Auckland, New Zealand

**Keywords:** autoethnography, collaborative writing, dialogic play script, time, COVID-19, online/offline compression

## Abstract

In this article, we “write-to” time from an autoethnographic perspective. Working intra-actively via a dialogic play script form, we collaboratively wonder about time during our experiences of COVID-19 as it relates to a compression of offline into online spaces. Presenting conversations we’ve had together over email, WhatsApp, and Google docs, with the reviewers of this Special Issue, and with scholarship, we foreground three main questions: What does time mean? How has our sense of time changed? And what is the link between these meanings and changes and the relationship between online and offline spaces?

time (mass noun) The indefinite continued progress of existence and events in the past, present, and future regarded as a whole—“travel through space and time.”

—Oxford English Dictionary

It’s Tuesday the 14th July—the time is 3.07 PM in Melbourne and 5.07 PM in Auckland. I—Brook—am sitting at my desk, enjoying a moment of Zoom silence, and beginning to rework the abstract Philippa and I submitted for this Special Issue in *Qualitative Inquiry*. I have 1 hour. Then I will swap toddler care with my husband, and probably end up trying to skateboard down our driveway while my 2.5-year-old races me on his balance bike. I’ll send the results of this hour’s worth of thinking to Philippa, who’s in Auckland. When I send it to Philippa, it will be past 6 in the evening in Auckland, and Philippa may not see it until the morning. I have no idea what contexts—online and offline—will frame where Philippa is when she looks at the thoughts from this hour, what type of device she will use to access it, how much time there will be between her viewing, reading, and going online to respond and engage with these thoughts, and what other personal and professional activities and responsibilities will compete with me, for Philippa’s time.

It’s 9.30 PM on Tuesday 14 July in New Zealand and I—Philippa—am sending my final text message of the day to Brook. We have exchanged eight texts and 14 voice messages within the last four and a half hours as we brainstorm the writing of this article together. My digital communication is always on, particularly since the government’s imposed lockdown has meant that my only connection with others outside of my house is via social distancing and the digital. The feelings of saturation with the digital and my reliance on it throughout the course of the day however are increasingly overwhelming—for work: online lectures, answering student emails, beaming into countless, relentless, Zoom meetings or, from a more personal perspective, online pilates classes, Zoom quiz nights with the family across the globe’s two hemispheres, finding amusement in crazy lockdown memes, or checking news for the latest tally of COVID-19 cases here and around the world. But, even now that my university has raised the green flag to return to campus (staff only, not students) the experience is eerie; it is a changed world. I use my iPhone to scan a QR code as I enter and depart every building so that my movements can be tracked, just in case I encounter a COVID-19 infected person. I am greeted by hand sanitizer stations as I go. The corridors echo with the sound of my footsteps. Hardly anyone in sight, with my colleagues preferring to work from home. I crave the physical presence of others—but, for now, my communication will remain mostly digital.

Observing the blackness of the evening through the window, I respond to Brook’s latest voice message. She delivered this in a whisper, presumably as not to disturb her son who is likely in bed. But like him too, I need to recover from the busyness of the day and I tell Brook I need more time, will “sleep” on our ideas and send my thoughts to her in an early morning email. While I believe I will now be offline, I carefully place my iPhone beside my bed, just in case.

*****

Seven years have passed since we first met at a summer school in Switzerland on research methods for scholars interested in digital discourse. Our shared interest, together with a desire and need to try to make sense of our own experiences of lockdown, led us to reconnect through participation in a project conducted via email and Facebook titled “Massive and Microscopic sense-making in the time of COVID,” launched by Annette Markham and Anne Harris (2020). Unlike our first face-to-face encounter, we’ve worked together on this project remotely, under two periods of staggered lockdown to make sense of the situation we find ourselves in. We are challenged with the simultaneous juggling of professional and personal demands, against a backdrop of global anxiety and uncertainty. We feel deeply unsettled, despite our privileged economic positions and relative job security.

In our voice mail exchanges we discuss how our engagement with offline and online spaces appears to have altered, and particularly our sense of the relationship between them, and we observe that this change greatly impacts our sense of time. *We feel we’re somehow always online and offline at the same time*, and this feeling is acute and relevant across our roles as academics (educators, researchers, and administrators), partners, and mothers (of a toddler—Brook—and of three grown daughters separated between London and New Zealand—Philippa).

In rekindling our friendship in this collaborative autoethnographic piece, we work to make sense of, what the title of the project has referred to in temporal terms as, “the time of COVID.” Gale and Wyatt’s (2009) “between the twos” approach to collaborative-writing-as-inquiry enables us to “write-to-it”—“‘it’ being the query or problem” (Gale & Wyatt, 2017). In this case, both the query and the problem, prompted through our diary entries in response to the project,^1^ lead us to ask: What does time mean? How has our sense of time changed? And what is the link between these changes, and the relationship between online and offline spaces?

Working intra-actively and following Wyatt and Gale’s (2018) dialogic play script form, we are, in their words, “transversally engaging and actively producing through the animation of a philosophy of the event” (p. 120)—producing to probe time, and the relationship between time and the lines (online/offline). In writing this piece, we’ve written, spoken, and thought collaboratively, and we try to indicate processes of initial authorship and subsequent reaction (Ellis et al., 2011) to each other. As Wyatt and Gale (2018) emphasize, “[w]riting to it is an act; it is about bringing concepts to life” (p. 123). Our attempts to write-to-time in the subsequent conversations we share here thus lay bare an act through which we bring to life ideas around “COVID-19 time” and the bleeding of online and offline contexts that frame and stem from trying to make sense of this time.

## Imposed Timings

Brook: It’s now August 10th. We’ve just spoken over WhatsApp about Melbourne being back in lockdown. It’s the most severe stage yet. And for the first time, childcare has shut. Everyone tells me just do your best. But I don’t think my best is enough. I feel squeezed in different directions. I feel like there’s not enough time to do anything well. I know others feel this way, too. I remember you telling me on Friday, that I shouldn’t feel guilty for prioritizing my son. I’m anxious because I had hoped that Stage 3 would lead to Stage 2, and not Stage 4.^2^ I’m trying to make sense of the changes to my life, like you are. I think many of these changes relate to the compression of time, and I’m becoming increasingly aware that this has an impact on where I am during the day. I feel like I’m both always online and always offline. I’m not sure this distinction makes all that much sense to me anymore.

This sets me wondering: Harvey (1989) tells us that “[i]ndividual biographies can be tracked as ‘life paths in time-space’” (p. 211). Listen to what he says next: “[. . .] beginning with daily routines of movement (from house to factory, to shops, to school, and back home again), and extending to migratory movements over phases of a life-span” (p. 211). But you and I are mostly doing all of this at home, online, like others who are privileged enough to be working from home. Perhaps, the fact that these movements have become more virtual and less physical affects how we are now experiencing time, and in turn, our very sense of self? I think what we’re suggesting in our many virtual conversations is that there’s a real link between changes in our individual biographies of time and the spaces in which we’re living under COVID-19. And that these spaces can’t really be distinguished as online or offline—they’re bleeding into each other, so that they’re both.

Philippa: Bleeding, yes b-l-e-e-d-i-n-g . . . . these letters roll off my tongue and percolate in the deepest corners of my mind. Like you Brook, I am only just beginning to realize the pain of lockdown in terms of time compression—this bleeding (perhaps even a hemorrhaging, though this may be too strong a word) of our online/offline selves. How wonderful metaphors are in times of chaos. They enable us to think in imaginative ways, to sift through the debris of our minds and search for meaning about ourselves and our world. Bleeding suggests pain and we visualize the fluidity of the online and offline as they merge, mix and mingle so that we no longer perceive them as binaries, but accept them as part of the pain of our “new” ways of being under lockdown.

I’ve always relished the digital age and the boundless opportunities to communicate with others instantly and across vast distances. But your wonderings about time compression strike a chord—they resonate with me. Our individual biographies both intersect and dance around each other based on our shared experiences, you in Melbourne and me in Auckland. I have struggled to free myself from anxiety and resentment about living a different life where my sense of time has become confused and clouded. Those taken-for-granted daily routines of going to the supermarket, teaching in a classroom, meeting friends in a cafe are now compressed and forced into a digital time warp that no longer allows me the pleasure to experience the physical world as I have known it, and still wish to know it.

Brook: I’m walking now during my brief time outside, my mask enveloping my nose and mouth. I am recording a voice message to send to you—it’s this message that I’m writing down for this paper now that I’m back on my computer, still online.

I wonder how to best make sense of this time confusion: our day to day lives have changed under COVID-19 and this is a change in the life biographies that we have been speaking about. I guess that’s also the idea of “the general temporal structure of consciousness” that Bourdieu (1977) points to and Atkinson (2019) discusses; our life biographies are different now whether we like it or not. These changes mean that we’re at home more than we were, we’re more physically distant than we were, and we’re online more than we were. And we experience this time largely as imposed, because it’s a direct result of a new clock. Like daylight saving, we must adjust the hands on the clock to fit a new way of being.

Do you remember us talking over WhatsApp about Atkinson’s (2019) definition of “imposed timings”?—the idea he develops in relation to Bourdieu’s (1977) *Outline of a Theory of Practice*, that there are perceptions and time that don’t come from our positions in particular fields, but are imposed from outside—like

[t]he schedule of public holidays, the imposition of time zones or daylight savings time, working time regulations, the timetables of schools or organisations one is not an effective agent within, opening times of businesses and even clock time itself are all prominent examples. (p. 959)

We’ve been told we must move in a particular way for work, for everything. And this is squeezing and pressing these many different activities together. It’s making me struggle and feel pressure.

Philippa: Yes interestingly while we bravely want to fight and eliminate this virus for the good of our nations and the world, as the lockdown cycles through periods of on-again/off-again/on-again with little advance warning, our opportunity to return to a life without imposed timings decreases.

I am feeling the pain. My life is a pressure cooker. One moment the valve unscrews allowing a blast of my pent-up frustration and anxiety with the global pandemic to escape as we are offered freedom again to get our hair cut or go to a restaurant with friends. The next moment the valve is quickly tightened, we’re forced back into our homes again under a new alert level, and I feel that accumulating pressure again as I struggle mentally and emotionally in teaching my students online, worrying about my elderly mother in isolation, or mourning the loss of the physical presence of my friends and colleagues.

Brook: More metaphors abound! This whole situation makes me feel like a frog. The ones that don’t notice they’re being cooked because the water is slowly getting hotter. You might see the funny side of this, but this “new normal” of imposed COVID-19 time is worrying me. We’re expected now, I think, during this second round of lockdown, to do more, because it worked the first time around. And this pressure cooker for you, this steady increase in the water temperature that slowly cooks me like the frog, epitomizes our struggle. Because we’re at home, all the demands from different fields are compressed.

I worry about how to work full-time and be a good mum. And in the absence of these physical routines that extend from and to our homes, it’s harder to demarcate chunks of the day from one another. We no longer have the physical movement to help us do so.

Philippa: I’m sitting at my desk at home right now, writing this passage on my laptop. Auckland is suddenly thrust back again into a second imposed lock down—deja vu! I see my neighbor Paul pulling up in his faded blue SUV in the driveway outside my window. He smiles and waves as he gets out of the car, noisily banging the door shut. I’m not used to seeing Paul on weekdays, I’m usually at work. It’s confusing. He belongs in my field of weekend time, where I chat to him over the hedge or pat his elderly labrador Ben who always looks at me with searching, soulful eyes. I notice this strangeness, but suddenly I’m drawn back to my computer screen as an email signals its arrival in my inbox advising that the forthcoming student winter graduation may be postponed. “More information to come” I’m told.

So, both our sense of space and time is being compressed, and this compression takes the form of an ever-increasing presence of the online. My body, aching from this uncomfortable “it-will-do” chair, remains motionless at my desk. Only my eyes move, shifting from Paul outside into the digital space of the email that demands my attention. I’m conscious I’m in two spaces at the one time—the physical and the online.

Brook: I remember during the project, I found myself writing about this very inability to separate being online from being offline. For me much of this is about no longer being able to separate my work-self from my mother-self. I feel the pressure to always be both. I guess I never imagined the digital would become so central to my life, by this large “thing” that’s happened. I’m not unfamiliar with being online, of course. I’ve often gotten support and networking through various online parenting groups. Some are local groups, like the one for mothers in the area where I live now. Others, like the breastfeeding and gentle sleep groups, are ones I joined first in Hong Kong and then in Australia as I tried to make sense of major changes in my life. But this level of being online is different. I’m on Zoom endlessly. I meet on Zoom. I teach on Zoom. I have social time on Zoom. And Facetime is the only way we reconnect with family and friends. I record myself trying to smile for my students in my videos, hoping my voice sounds engaging. Even when I’m not on Zoom, I’m on Zoom. Did you know I have started to hear people speak as I read their messages? Their voices appear in my head, and I hear the way they speak emerging from my memory of when we were last on Zoom.

So now I’m thinking that this struggle I’m experiencing is largely to do with the fact that the need to be both work-self and mother-self at the same time is being imposed.

Philippa: While the two of us are immersed in different stages of family life (you Brook, with the demands of a young child and me an “almost” empty nester with the return home of our 23-year-old who helps cook, clean, make us laugh and teaches us to “Zoom”), it is this permeation, the feeling of imposition from the outside as a side-effect of COVID-19 timing, that are inherent in my project diary entries too. You talk emotionally of this imposition and the struggle when your pleasure of using the digital for parenting support and connection (so important!) has been hijacked as it becomes a “requirement” in other activities pervading your everyday life. This changes how you feel about your new circumstances because this is beyond your control. For both of us, the constant demands of “being there” and “being accessible” for online lectures, for Zoom meetings, with the pressure to look “respectable” through the lens of the camera etc. whatever the time of the day—this is what binds and squeezes us. We are bound and squeezed, squeezed and bound . . . into a state of confusion as we resist this imposition on our home life that we regard as sacred and closely protect.

Atkinson (2019) refers to the pacing of field events, in particular “work *intensification*” (his italics) and the increased pressure to “be more productive, to work quicker, with more and tighter deadlines,” which he ties closely with the affordances of communications technology (p. 957). The result: different fields, such as work, have greater spatial reach which can be achieved at speed through the internet. In our case, this sums up exactly the pain we are experiencing in terms of the bleeding of the online/offline in our fields of work and domestic life.

I feel this right now. I’m sitting in casual jeans at my laptop (not my usual work attire, but who cares?). Today’s list of work tasks lies scribbled on a piece of paper to my left. It’s almost lunchtime and I’m feeling drained. Shortly I will call out to my youngest daughter, once she has finished that work Zoom meeting I hear her conducting from the dining room downstairs—“shall we meet for lunch?” When we stand together at the open fridge door, as we do most days, our eyes will search for something appealing to eat, but without much success. We are too busy working from home to stand in the supermarket queue, avoiding other shoppers with their eyes peeking over their paper-thin masks. Online shopping has a 3-week delay. That’s way too long. Instead we will reach to the back of the freezer for that solidified, pumpkin soup that nestled there for some time. Then it’s back to my laptop.

Brook: I think what we’re feeling here is the “friction of distance” (see also Harvey, 1989): We’re trying to find an appropriate response to the physical distance COVID-19 has created, and needing a huge amount of energy, effort and time to do so. Because, as you say, we’re not just doing this in our work lives, but also socially. And because we’re needing to do much of it online, there’s no real separation anymore: from me writing a work email on my phone, while trying to engage with my toddler at the same time at play. I refuse to see this as the joy of multi-tasking. I’d rather just be with him. But here I sit, on the chair in his playroom, and he’s clambering onto me, asking me to hold the book he wants me to read, not in one hand, but in two hands. Where can I hold my phone, to check and respond to emails? I slide it onto the armrest, and actually manage to type a message while reading aloud at the same time. The perfect online/offline pendant to being able to tap my head and rub my tummy at the same time. Something to be proud of though . . . I’m not sure.

## Time Across the Lines

Brook: In bringing together imposed COVID-19 timing and time “binds,” “squeezes,” and “crunches” (Atkinson, 2019) with compression, I feel like we’re almost suggesting that the online is becoming a “figure” to the offline’s “ground.” I’m thinking here of that vase-face image. You know the one, right? Where one person sees a vase but another can only see a face. Jones (2004) writes about this in a great paper where he argues that online practices don’t only take place online. The contexts are virtual and material. He also says that the online needs to be rethought of as an extension to the offline world, and not as separate. Something online users have known for some time, but scholars have been slower to respond to.

I think we’re pretty used to this idea of the online being an extension of the offline. But I think what we’re demonstrating through the sharing of our experiences is that many offline practices have now become compressed *into* online ones.

My husband asked me last night when I was talking to him about our paper whether I might be part of a last generation that sees things this way—who might see this compression as challenging, given that many generations have gone through the normalization of massive technological changes (telegraph, radio, TV, phone, etc.) before us.

Philippa: When I talk to my students about life “before” the internet, they struggle to understand what I mean because the digital is so ingrained in the normality of their daily lives. Orgad (2009) argues that the online and offline have long been viewed as separable and separate. But, I believe that over time this perception is changing, especially when digital natives, like my students, regard any notion of separation as irrelevant. It’s a matter of perspective—like the vase-face image—when people might see one or the other, possibly both alternatively, but not at the same time. It also reminds me of the photo that went viral on the internet of the dress that looked blue to some but silver to others. Curiously I was able to see both which was a very weird feeling. But it shows that there is more than one way of interpreting the world depending on who we are, our generational positioning, our ideologies and worldviews.

I guess this raises the question as to whether we belong to an older (or should I say more mature?) generation trying to resist the bleeding of the online/offline because we see it as an invasion, disrupting the world as we know it. I remember when my oldest daughter at the age of 14 got her first mobile phone back in the early 2000s. I laid down the law—“no phone in the bedroom at night!” She was distraught and it was only when another parent told me (politely) that I needed to “stress less” because this was the new world of communication for young people, that I conceded. And I acknowledge that I, too, now religiously take my phone to bed with me at night. But this was a world I came to accept over time.

COVID-19 has imposed a different world on us, and I wonder Brook, for your young son, whether he will understand when he is older our talk of online/offline.

Brook: I think my son sees my phone as almost a physical part of me. It’s the place where strange people’s voices come from when I play my colleagues’ imessages or WhatsApp messages aloud; I type into it at random in the middle of reading him a book; we use it to call his grandparents, propping it up so they can play trains together; we look at photos and use it to create memories; and, for him, most importantly, it’s magic because it’s where Peppa Pig comes from.

I think it is a question of continuity and permanence: How much has changed, or how much we think has changed, and how different these thoughts are depending on who’s doing the thinking.

For me, there’s definitely a tension between what I thought was possible before and what I’m experiencing now in COVID-19 time. A colleague of mine (thanks Howie Manns, personal communication!) told me about E.M. Forster’s *The Machine Stops* just yesterday. Perhaps you know it already? It’s a science fiction short story, initially published in 1909. According to Wikipedia (n.d.a):

[t]he story describes a world in which most of the human population has lost the ability to live on the surface of the Earth. Each individual now lives in isolation below ground in a standard room, with all bodily and spiritual needs met by the omnipotent, global Machine. Travel is permitted, but is unpopular and rarely necessary. Communication is made via a kind of instant messaging/video conferencing machine with which people conduct their only activity: the sharing of ideas and what passes for knowledge.

For me so much of COVID-19 has felt surreal, like I cannot grasp what’s happening, but am desperately trying to do so. But I’m continuously also reminded that this is part of a larger narrative. If Forster could imagine in 1909 this kind of world in which instant messaging is dominant because people cannot interact face-to-face, then what larger story is this online COVID-19 compression part of?

Philippa: The larger story of online COVID-19 compression? Perhaps this is what we invoked with our final collaborative moving image piece in response to Prompt #21 for the *Massive and Microscopic sense-making* project without even realizing it.^3^ Perhaps this is the road we were heading down all along—it’s just taken this collaborative writing-to-time to bring us to this point in our questioning, of the bleeding of the online/offline, to see where it fits in a greater narrative.

**Figure 1.**
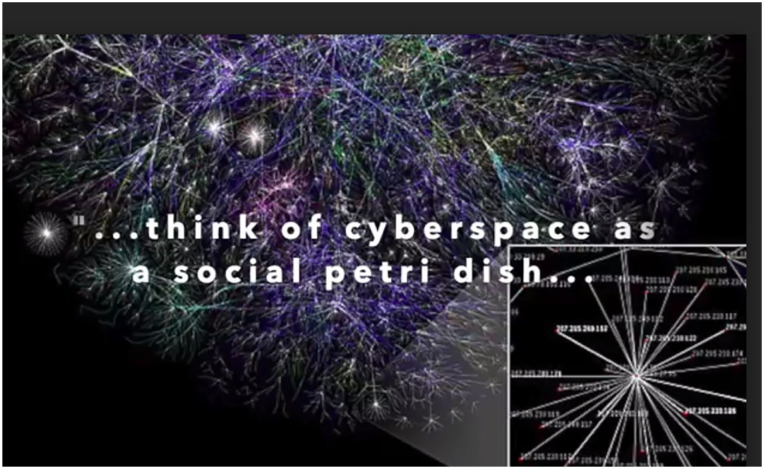
Screenshot of the beginning of our creative prompt by Brook Bolander, Philippa Smith, & Fiona Stirling (2020). (Background image of Opte Project internet map 2006, sourced through Wikipedia (n.d.b) and reproduced under Creative Commons Attribution 2.5 Generic license, https://creativecommons.org/licenses/by/2.5/deed.en).

I guess it was my preoccupation with Benedict Anderson’s (1991) “imagined communities” and the creation of nation states that drew me back to reading Howard Rheingold’s (1993) book *The virtual community: Homesteading on the electronic frontier* as a stimulus for our creative project. I thought that Rheingold, one of the first to write about personal experience of a *virtual* community in the early 1990s, might inspire understandings about our experience of online and offline during COVID-19 time. His interpretation of virtual community caught my eye (and which we used in the opening of our video, see Figure 1):

Think of cyberspace as a social petri dish, the Net as the agar medium, and virtual communities, in all their diversity, as the colonies of microorganisms that grow in petri dishes. Each of the small colonies of microorganisms—the communities on the Net—is a social experiment that nobody planned but that is happening nevertheless. (p. 521)

I’ve always marveled at the intricate connection between time and space (nostalgic aside: *Lost in Space* and *Time Tunnel* were two of my favorite childhood TV programs)—and Rheingold’s description here is trying to make visible the invisible for us—and once again, a metaphor helps this visualization. Something that is so massive that it is described in terms of cyber-“space” is simply reduced, condensed, into small colonies of microorganisms growing on a petri dish.

Is there a connection here with COVID-19 time? Can we take these words from a quarter of a century ago, Rheingold’s command for us to “think” how we are part of a social experiment because we use the internet? I marvel at the irony that both virtual communities and the virus might only be made visible through the magnification of the lens of a microscope. The massive and the micro become intimately entwined in our imaginings of both cyberspace and of COVID-19. They are related in the sense that they have become significant in our lives, whether we like it or not. But the keywords in this quote are that the Net is “a social experiment that nobody planned but that is happening nevertheless.” I wonder whether we might see COVID-19 under these same terms, to see we are part of a social experiment that nobody planned; that we have had no choice in becoming a part of. I wonder whether this is an example of what Wyatt and Gale (2018) refer to as “clouding” where writing or even thinking collaboratively can take us “into what we don’t know” (p. 124) or perhaps don’t expect.

Brook: Like you, I’m struck by Rheingold’s wording, and the scientific register he employs. I remember when we were working on this project, feeling intrigued by there being both change and continuity surrounding ideas of community and particularly the online/offline relationship. I also remember feeling sad and angered by the new connotations the phrase “social experiment” took on, when the quote was recontextualised and mashed into a kind of COVID-19 temporal and mental schema.

It was this that led me to compose the following text to include in our video, which we recorded ourselves reading aloud in separate recordings, before Fiona merged them to create the semblance of a layering of words across space and time for our final output.

More than 25 years later, we are forced to recognize that this dish is not transparent. We are forced to recognize that we are not all biologists. We are forced to recognize that the experiment hurts. We are forced to recognize that it hurts some far more than others. We are forced to recognize that even while the dish may seem shallower, more porous, offline social distancing both reduces while reinforcing and reifying boundaries between online and offline. What, if we look at this social petri dish now, under COVID-19, under the bleeding world, is the dish? Who are the colonies of microorganisms? How small are they? Can we see them? Can we see ourselves in them?
Philippa: So is this the bigger picture that our “writing-to-it” has been moving toward, our lightbulb moment? We have lifted stones, navigated the literature, searched our souls for answers. It is hurting us and we have attempted to make sense in this paper of the imposition of compressed time felt through the bleeding together of the online and offline under lockdown, the invasion of the digital into parts of our lives we wish to be kept separate. Is this something that we should adapt to or can we use it to help future generations?

Perhaps, we are the generation of resistance to offline/online compression while upcoming generations may not even notice. My mother and her family came from Vienna to New Zealand as refugees just prior to the outbreak of World War II. This was a different world for them, but they had to adapt and build a new life in New Zealand. I know this comparison may seem extreme and I struggle with this, but I use it to explain that as children we too were a new generation growing up in a different time from our parents. They were the ones who had to adapt, while we just lived it. Maybe our lockdown experience should be turning our focus on envisaging a new world and better future for our children, post-COVID-19.

## Emerging Through the Time Warp

In this article we have tackled the notion of time, and in particular a bleeding of online and offline spaces, which has greatly affected us during repeated, yet staggered episodes of COVID-19 lockdown. Following the autoethnographic approach of “writing to it” by way of a dialogic film script form proposed by Wyatt and Gale (2018), we have tread a path where the two of us have reflected and shared our experiences in two different countries through asynchronous communication of text, email, voice messages, and collaborative Google documents. We have drawn on our own field diaries from the 21-day project (Markham & Harris, 2020) where we sought to “wonder about” and make sense of our brave, new world through a focus on the digital and its intersection with the changing notion of time. We’ve thrown ideas back and forth between us, looked to the scholarly literature for guidance and insights, and responded to reviewers’ comments.

For Gale and Wyatt (2017), the term “wonder” draws attention to the “potential” collaborative writing has to “take us [. . .] somewhere different” (pp. 355–356). In this sense, it reminds us of the importance of “conversation” for the development of ideas and the creation and dissemination of knowledge (Baym & Markham, 2008; Heller et al., 2018), despite the fact that such conversations about method typically remain outside of published work.

We are not surprised that other scholars in this themed issue have eloquently referenced time amid their COVID-19 probings: Annette Markham’s counting of time in daily patterned activities, Annabelle Sreberny’s observation of the paradox of time seemingly flying by and slowing down simultaneously during lock down, and Stephanie Shelton’s exploration of the Doomsday Clock and the Peace Watch Clock as a way to interrogate the new “messiness” of COVID-19 time, to mention a few. But for us as a linguist and a critical discourse scholar with interests in digital communication, our focus has been on the compression of the offline into the online that we have felt so strongly; and in writing this piece, we’ve tried to bring to life how difficult it’s become for us to demarcate the time dedicated to work and home life. We’ve toyed a lot with trying to understand this imposition of time and what seems to increasingly be the “new normal.” Much of this discussion is about the workplace and the economy. But, of course the digital is a huge part of this.

As Wharf (2011) writes, it’s

[o]nly when we explicate how time and space are continually transferred [that] we can avoid the simplistic arguments that lead to erroneous conclusions of a “flat world” (Friedman 2005), the “end of geography” (O’Brien 1992), or the “death of distance” (Cairncross 1997). (p. 435).

So, we get that “[t]he distinction between the online and the offline has been constitutive of the understanding of the internet from the earliest days of internet research” (Orgad, 2009, p. 36). But the shift in perspective that we have identified realizes the bleeding of the offline into our online spaces, and acknowledges a growing focus on how users exploit the affordances of an increasingly multimodal set of technological resources to engage, communicate, and negotiate ideas and relationships in their daily lives. Intersecting with this change in viewpoint is a steady complicating of context that adds layers: of social facets (Herring, 2007), of participants (Dynel & Chovanec, 2015; Marcoccia, 2004), and of setting or “Umwelt” (Jones, 2004; Bolander, 2019; Bolander & Locher, 2020). From this vantage point, social actors and their modes of communication are no longer understood as if in a “virtual vacuum” (Jones, 2004, p. 21). COVID-19 brings this realization into sharp focus, and the offline-into-online compression turns it on its head.

*****

Brook: I’m sitting here now, thinking about time and space transfer. It’s 1.25 PM on Thursday 13th August. I’m transferring these words onto a page in a Google doc, that you can see at the same time, or could if you were online right now. I think you’ve gone for a 15-minute walk. Some movement that may take you offline. I’m not sure, because perhaps you have your phone with you? In a moment I’ll leave you a voice message on WhatsApp to tell you that the paper’s back to you. I won’t move far though. Upstairs to the playroom, or to our driveway, but I’ll have my phone on me, so I’ll stay online.
Philippa: The sun is sparkling on the waters of the Waitemata Harbor as I meander along the waterfront, trying to clear my head after a day of intense writing on my laptop. I sidestep an oncoming runner wearing a face mask, our eyes fail to connect. At the same time the phone in my pocket signals your incoming voice message. You tell me that after my next edits you’ll have one more day to work on the google document before handing it back to me on Saturday. At that instant I stop in my tracks, I push the pause button and ponder—is Saturday tomorrow or the day after? I have lost all sense of time.

## Notes

1. In referencing our diary entries or prompts, we are referring to our engagement across 21 days with a series of prompts issued by Markham and Harris (2020) in connection with the massive and microscopic sense-making project (see also Markham et al., 2020)

2. In Australia, Stage 4 is the most severe stage of lockdown.

3. Prompt 21 called for a final collaborative performance “to move past simply describing what’s presently happening, to consider what these particularities of the situation mean, at a broader, cultural level. What is it teaching us, how does our embodied experience and knowing connect to larger issues, other scales and levels of knowing?” (Markham et al., 2020)

## References

[bibr1-1077800420962476] Anderson B. (1991). Imagined communities: Reflections on the origin and spread of nationalism (Rev. and extended ed.). Verso.

[bibr2-1077800420962476] Atkinson W. (2019). Time for Bourdieu: Insights and oversights. Time & Society, 28(3), 951–970.

[bibr3-1077800420962476] Baym N. Markham A. (2008). Introduction: Making sharp choices on shifting ground. In Markham A. Baym N. (Eds.), Internet inquiry: Conversations about method (pp. vii–xix). SAGE.

[bibr4-1077800420962476] Bolander B. (2019). Social media research. In Östman J. A. Verschueren J. (Eds.), IPrA handbook of pragmatics (pp. 31–48). John Benjamins.

[bibr5-1077800420962476] Bolander B. Locher M. A. (2020). Beyond the online offline distinction: Entry points to digital discourse. Discourse, Context & Media, 35, 100383. 10.1016/j.dcm.2020.100383

[bibr6-1077800420962476] Bolander B. Smith P. Stirling F. (Producers). (2020). Homesteading Across Time. [video]. https://drive.google.com/file/d/1jfpPMJXWJZH1NzXHPoobEfsTGk4HlNZk/view?usp=sharing

[bibr7-1077800420962476] Bourdieu P. (1977). Outline of a theory of practice. Cambridge University Press.

[bibr8-1077800420962476] Dynel M. Chovanec J. (Eds.). (2015). Participation in public and social media interactions. John Benjamins.

[bibr9-1077800420962476] Ellis E. Adams T. Bochner A. (2011). Autoethnography: An overview. Forum: Qualitative Social Research, 12(1), Article 10.

[bibr10-1077800420962476] Gale K. Wyatt J. (2009). Between the two: A nomadic inquiry into collaborative writing and subjectivity. Cambridge Scholars.

[bibr11-1077800420962476] Gale K. Wyatt J. (2017). Working at the wonder: Collaborative writing as method of inquiry. Qualitative Inquiry, 23(5), 355–364.

[bibr12-1077800420962476] Harvey D. (1989). The condition of postmodernity. Blackwell.

[bibr13-1077800420962476] Heller M. Pietikäinen S. Pujolar J. (2018). Critical sociolinguistic research methods: Studying language issues that matter. Routledge.

[bibr14-1077800420962476] Herring S. C. (2007). A faceted classification scheme for computer-mediated discourse. Language@Internet, 4, Article 1. https://www.languageatinternet.org/articles/2007/761

[bibr15-1077800420962476] Jones R. H. (2004). The problem of context in computer mediated communication. In LeVine P. Scollon R. (Eds.), Discourse and technology: Multimodal discourse analysis (pp. 20–33). Georgetown University Press.

[bibr16-1077800420962476] Marcoccia M. (2004). On-line polylogues: Conversation structure and participation framework in internet newsgroups. Journal of Pragmatics, 36(1), 115–145.

[bibr17-1077800420962476] Markham A. Harris A. (2020). Massive and microscopic sensemaking call. Call for Expressions of Interest. https://futuremaking.space/call-for-participation/

[bibr18-1077800420962476] Markham A. Harris A. Luka M. E. (20XX). Massive and microscopic sensemaking in times of COVID-19. Qualitative Inquiry.

[bibr19-1077800420962476] Orgad S. (2009). How can researchers make sense of the issues involved in collecting and interpreting online and offline data? In Markham A. Baym N. (Eds.), Internet inquiry: Conversations about method (pp. 33–53). SAGE.

[bibr20-1077800420962476] Rheingold H. (1993). The virtual community: Homesteading on the electronic frontier. Addison-Wesley.

[bibr21-1077800420962476] Wharf B. (2011). Excavating the prehistory of time-space compression. Geographical Review, 101(3), 435–446.

[bibr22-1077800420962476] Wikipedia. (n.d.a). The machine stops. https://en.wikipedia.org/wiki/The_Machine_Stops

[bibr23-1077800420962476] Wikipedia. (n.d.b). The Opte Project. https://en.wikipedia.org/wiki/Opte_Project

[bibr24-1077800420962476] Wyatt J. Gale K. (2018). Writing to it: Creative engagements with writing practice in and with the not yet known in today’s academy. International Journal of Qualitative Studies in Education, 31(2), 119–129.

